# The impact of non-response bias due to sampling in public health studies: A comparison of voluntary versus mandatory recruitment in a Dutch national survey on adolescent health

**DOI:** 10.1186/s12889-017-4189-8

**Published:** 2017-03-23

**Authors:** Kei Long Cheung, Peter M. ten Klooster, Cees Smit, Hein de Vries, Marcel E. Pieterse

**Affiliations:** 1 CAPHRI Care and Public Health Research Institute, Health Services Research, Maastricht University, Duboisdomein 30, 6229 GT Maastricht, the Netherlands; 2 Psychology, Health & Technology, University of Twente, Enschede, the Netherlands; 3 CHS of Twente, Enschede, the Netherlands; 4 CAPHRI Care and Public Health Research Institute, Health Promotion, Maastricht University, Maastricht, the Netherlands; 5 Psychology, Health & Technology, University of Twente, Enschede, the Netherlands

**Keywords:** Non-response, sampling bias, health behaviour, recruitment, adolescents

## Abstract

**Background:**

In public health monitoring of young people it is critical to understand the effects of selective non-response, in particular when a controversial topic is involved like substance abuse or sexual behaviour. Research that is dependent upon voluntary subject participation is particularly vulnerable to sampling bias. As respondents whose participation is hardest to elicit on a voluntary basis are also more likely to report risk behaviour, this potentially leads to underestimation of risk factor prevalence. Inviting adolescents to participate in a home-sent postal survey is a typical voluntary recruitment strategy with high non-response, as opposed to mandatory participation during school time. This study examines the extent to which prevalence estimates of adolescent health-related characteristics are biased due to different sampling methods, and whether this also biases within-subject analyses.

**Methods:**

Cross-sectional datasets collected in 2011 in Twente and IJsselland, two similar and adjacent regions in the Netherlands, were used. In total, 9360 youngsters in a mandatory sample (Twente) and 1952 youngsters in a voluntary sample (IJsselland) participated in the study. To test whether the samples differed on health-related variables, we conducted both univariate and multivariable logistic regression analyses controlling for any demographic difference between the samples. Additional multivariable logistic regressions were conducted to examine moderating effects of sampling method on associations between health-related variables.

**Results:**

As expected, females, older individuals, as well as individuals with higher education levels, were over-represented in the voluntary sample, compared to the mandatory sample. Respondents in the voluntary sample tended to smoke less, consume less alcohol (ever, lifetime, and past four weeks), have better mental health, have better subjective health status, have more positive school experiences and have less sexual intercourse than respondents in the mandatory sample. No moderating effects were found for sampling method on associations between variables.

**Conclusions:**

This is one of first studies to provide strong evidence that voluntary recruitment may lead to a strong non-response bias in health-related prevalence estimates in adolescents, as compared to mandatory recruitment. The resulting underestimation in prevalence of health behaviours and well-being measures appeared large, up to a four-fold lower proportion for self-reported alcohol consumption. Correlations between variables, though, appeared to be insensitive to sampling bias.

## Background

When monitoring health indicators and risk behaviour among adolescent populations, it is important to understand the magnitude of selective non-response and the impact this may have on the prevalence estimates. As described by Berg [1]: “non-response bias refers to the mistake one expects to make in estimating a population characteristic based on a sample of survey data in which, due to non-response, certain types of survey respondents are under-represented” (p. 3). It seems that non-response bias is the rule rather than the exception in epidemiological surveys, and this is long recognised [2]. Literature on non-response bias through mailed surveys shows that non-response bias is a serious concern in survey studies [3, 4].

Selective non-response may be associated with general characteristics of the study population. Previous studies have shown that female, older individuals, and individuals with higher education levels are more prone to return postal questionnaires [5, 6]. In such cases biased prevalence estimates are often corrected by controlling for these demographic variables or by estimating weighted proportions [7]. However, selective non-response may also be due to the actual outcome variables of interest. Studies generally show that respondents in health surveys report better health status and more positive health-related behaviours than non-respondents, including self-rated health and chronic diseases, smoking, physical inactivity, obesity, [5, 8, 9], lower alcohol consumption [10–12], better mental health, better subjective health status, more positive school experiences [13–25], and less risky sexual behaviour [16] than non-respondents. These findings indicate that people with poorer health tend to avoid participating in health surveys. While there are many factors that are important in ensuring the generalisability of findings in health studies, unbiased subject sampling may be paramount. Due to subject self-selection, research that is dependent upon voluntary subject participation is particularly vulnerable to sampling bias [26]. Respondents whose participation is hardest to elicit are likely to report more risk behaviour [27, 28]. In spite of this, the literature on the methodological implications of non-response due to sampling methods seems rather limited, and pertaining to adolescent populations in particular [17, 18, 24, 27]. Therefore, this study investigates the impact of non-response bias on prevalence estimates among adolescents, by comparing data gathered through voluntary sampling (with a high non-response rate) with data gathered through a mandatory sampling strategy (with a high participation rate).

As the validity of prevalence estimates within a population may be affected by non-response, this may also apply to analyses of between-variable associations within such datasets. For example, adolescent research has shown that various health risks appear to cluster in individuals [29, 30], presumably the result of shared underlying distal determinants like low self-esteem [31] or adverse personality traits [32]. Therefore, when studying the causal mechanisms underlying adolescent health risk behaviour by analysing co-variates of these behaviours, it is conceivable that these analyses may be confounded by selective non-response [8]. In other words, it seems warranted to investigate whether non-response bias may, indirectly, moderate associations among health-related variables. Although a non-response bias in itself cannot be a true moderating variable, it may be considered as a latent moderator that represents effects of true moderators that in turn are affected by non-response. Examples of such moderators within the field of substance use research are demographic characteristics. Studies indicate that demographics may moderate associations between tobacco consumption on the one hand, and for example alcohol consumption, school experiences, mental health, subjective health status on the other. Similarly, associations between alcohol consumption and school experiences, mental health, and subjective health status may be affected by demographic variables [33–40]. For example, gender differences were found in patterns of association between substance use and mood disorders [33], and for the association between tobacco consumption and drinking [36, 41]. Summarizing, this evidence implies that a non-response bias may affect the demographic composition of a sample [5, 6], and these demographics in turn are known to moderate associations between other health-related variables. Similarly, this may also apply to other mechanisms through which a non-response bias may invalidate between-variable associations in epidemiological research.

In order to enhance our understanding of non-response bias in public health monitoring of adolescents, this study first aims to identify whether there are systematic differences in prevalence estimates between two similar samples but with different rates of non-response due to sampling strategy. Biases in prevalence estimates are tested for both demographic and health characteristics, in two ways: by comparing the observed rates in both samples with the estimates known from available population statistics, and by testing the differences between both samples directly. Second, as it is conceivable that due to a non-response bias associations between risk factors will be confounded, this study also examines sampling method (mandatory recruiting with high response rate vs. voluntary recruiting with low response rate) as a latent moderator of associations between health related variables within subjects.

## Methods

### Sampling methods

Seven Community Health Services [CHSs] in the eastern part of the Netherlands collaborated with Maastricht University on the project named E-MOVO, a Dutch acronym for Electronic Monitor and Health Education [42]. E-MOVO is an electronic monitoring instrument, aimed at providing insight into health of adolescents of the 8th and 10th graders of secondary education. Whereas in most regions participation for adolescents at participating schools was mandatory, regions had the option to choose another sampling method. We used the results of two regions which used two different ways of sampling. In the mandatory sample (region Twente) sampling occurred mandatory and adolescents were recruited via secondary schools. Students in participating schools were instructed to complete the online questionnaire during a single class session (approximately 45 min) [43]. In the voluntary sample (region IJsseland) the adolescents were recruited voluntarily and were invited via a postal mailing to their home address, containing a hyperlink and personal code to the online questionnaire.

Non-response bias in the mandatory sample is considered minimal, as non-participation occurs in clusters (i.e. schools and classes) instead of the individual level. Each school in the region was invited to have all classes participate. There were several schools that did not participate at all, and some participating schools did not include all classes, due to practical reasons such as scheduling difficulties and lack of computer rooms. Therefore, we assume that there is minimal non-response bias in the data of the mandatory sample at the individual level. In contrast, due to higher non-response in the voluntary sample, it is likely that there is more non-response bias compared to the mandatory sample, as non-respondents here may differ in several characteristics from respondents.

An important requirement for the purpose of this study is that both populations from which the two samples were recruited are indeed comparable. Both regions are geographically adjacent, and similar with respect to socio-economic and urbanisation characteristics. With regard to risk behaviour prevalence, interregional comparability can be verified with two Dutch data resources on alcohol and tobacco consumption. In both resources data were collected across all regions with a standardized recruitment strategy and questionnaire, allowing direct interregional comparisons without a differential bias due to non-response. First, in the Health Monitor of 2012, with a representative sample of Dutch adults of 19 years and older, smoking prevalence was estimated at 23.9% in Twente and 22.0% in IJsselland [44, 45]. Weekly prevalence of heavy drinking (consuming 5 or more standard units on a single day at least once a week) was estimated at 9.2% in Twente and 8.7% in IJsselland [44, 45]. Second, the Dutch Health Survey with a representative sample of Dutch individuals of 12 years and older, identified the percentage smokers in 2008 at 32.3% in Twente and 29.8% in IJsselland. Hazardous drinking prevalence, defined in this study as either heavy drinking or exceeding moderate drinking levels (≥14 units a week for females and 21 units for males), was estimated in 2008 at 20.7% in Twente and 19.8% in IJsselland [46]. In general, available national data show that both regions included in this study show negligible differences in alcohol consumption, and a small difference in smoking prevalence. Although these data could not be specified for adolescents, in the case of the Dutch Health Survey adolescents of 12 year and older were included in the estimates. Nevertheless, it seems reasonable to assume that the magnitude of interregional differences found among adults may also apply to the adolescent populations of these regions.

### Participants

In the mandatory sample, the CHS of Twente was involved in recruiting schools in the 2011 study and maintained contact with its 14 municipalities within the region. All 59 secondary schools were approached, from which 39 participated in the E-MOVO study of 2011. The research team of E-MOVO informed the municipalities via e-mail about the study. The CHS of Twente informed each municipality and recruited schools within the community by sending an information sheet. Within participating schools informed consent was obtained from parents via an opt-out procedure. In the voluntary sample, the CHS of IJsselland selected a random sample of youngsters between the ages of 12 and 23, stratified on all municipalities in the region. For comparison of the regions, only the ages from 13 through 16 were included. Informed consent was obtained by sending a postal mail to the parents with an information sheet and the invitation for their child to participate.

### Measures

All matching items between the two surveys (Twente and IJsseland) were analysed. Measures were based on self-reports which have been shown to be reliable regarding tobacco, alcohol, and other drug use among adolescents [47, 48].

#### Demographics

Gender, age (in years), and education (11 options in Twente, 15 options in IJsselland) were assessed. For analytic purposes, education was dichotomised into low (“preparatory middle-level vocational education”) or high (“higher general continued education”/“preparatory scholarly education”).

#### Tobacco consumption

Participants were asked how often they smoked at present (0 = not at all; 1 = less than once a week; 2 = at least once a week, 3 = but not daily; 4 = every day). As previous studies reported whether or not youngsters smoke daily and due to violation of the linearity assumption, tobacco consumption was dichotomised into ‘daily smoker’ and ‘non-daily smoker’ [49, 50].

#### Alcohol consumption

Alcohol consumption was operationalised with three items. Participants were asked whether they had ever consumed alcohol (yes; no), how often they had had alcohol in their lives, and how often they had consumed alcohol in the past four weeks (0; 1; 2; 3; 4; 5; 6; 7; 8; 9; 10; 11–19; >20 times). As multiple reports mention whether youngsters had or had not consumed alcohol in the past four weeks [49, 50] and due to violation of the linearity assumption, alcohol in the past four weeks was dichotomised (yes/no).

#### Mental health

The Strengths and Difficulties Questionnaire [SDQ] is a behavioural screening questionnaire for children aged 4–16 years [51, 52]. The SDQ consists of 25 items and measures five scales of five items each (i.e. emotional symptoms, conduct problems, hyperactivity-inattention, peer problems, and prosocial behaviour). It has been extensively validated in many countries [53, 54]. The internal consistency (Cronbach’s alpha of .64), test-retest stability (except for the prosocial behaviour subscale (.59), all intraclass correlation coefficients were above.70), and parent-youth agreement of the various SDQ scales have been found acceptable [54]. To estimate the ‘probability for any behavioural problems from the SDQ scores, a modified version of Goodman’s algorithm [51] was used for the total score. Based on the algorithm, the probability of a psychiatric disorder was calculated as ‘1 = unlikely’ (0–15), ‘2 = possible’ (16–19), and ‘3 = probable’ (20–40) [51].

#### Subjective health status

One item was used to measure the subjective health status, consistent with other studies (e.g. DeSalvo, Bloser, Reynolds, He, & Muntner, 2006 [55]) Individuals were asked how they perceived their health in general (1 = very good; 2 = good; 3 = neutral; 4 = not good; 5 = poor).

#### School experiences

Participants were asked with one item how they experienced school (1 = great fun; 2 = fun; 3 = neutral; 4 = not fun; 5 = dreadful).

#### Sexual behaviour

In order to measure sexual behaviour one item was used [56]. Individuals were asked whether they had ever had sexual intercourse with someone (1 = never; 2 = once; 3 = couple of times; 5 = regularly).

### Statistical analyses

First, for both samples we examined whether the observed distribution of demographics deviated from the expected distribution in the population. For gender, a one sample t-test was performed. For the distribution of age and education level we provided descriptive comparisons of the mean age and education level (high vs. low) of the samples to the population estimates available to the best of our knowledge. Statistical tests were not performed with these demographic variables as the reliability of these estimates was lower than for gender.

Second, tests were performed of differences between both samples. For demographic characteristics, an independent samples *t*-test was used for age, and Pearson χ^2^-test for gender and education level (high vs low). To examine whether the samples differed on health-related variables, we first conducted univariable logistic regression analyses for each health-related variable of interest as independent variable and sampling method as dependent variable (mandatory sample Twente =0, voluntary sample IJsselland =1). Although, theoretically, sampling method would be considered as the independent variable, this was reversed in these analyses to allow a uniform analysis technique to be used for all health-related variables, regardless of the different measurement levels of these variables.

For the logistic regression analyses we checked the linearity assumption for non-binary variables (i.e. sexual intercourse, subjective health, school experiences, tobacco consumption, alcohol in past four weeks, lifetime alcohol consumption, and SDQ). Except for SDQ, alcohol in past four weeks, and tobacco consumption, variables did not violate the linearity assumption. To solve this issue, these three outcome measures were recoded into binary (tobacco consumption: 0 = no daily smoker, 1 = daily smoker; alcohol past four weeks: 0 = no, 1 = yes) or three-level (SDQ: 1 = unlikely, 2 = possible, 3 = likely). Further, to examine whether the differences in health characteristics between the samples could be explained by differences in demographic characteristics, all multivariable logistic regression analyses were repeated, with demographics (i.e. age, gender, and education) added as covariates. Intercorrelations were checked to test for collinearity between the health-related variable and demographic variables entered into the model. No signs of collinearity issues were found among the independent variables with all tolerance levels above 0.1 [57] and VIF values below 10 [58].

To examine moderation effects of sampling bias on associations between health related variables within subjects, an interaction term was computed for sampling method with tobacco consumption. Then interaction analyses were performed using logistic regression analysis according to the procedure by Baron and Kenny [59], with tobacco consumption, sampling method, and the sampling*tobacco use interaction term entered as independent variables. As independent variables the following health variables were tested in consecutive models: mental health, subjective health status, and school experiences. The same procedure was followed for tobacco consumption, alcohol consumption, and alcohol in past four weeks as dichotomous dependent variables. Due to the large sample size in this study a significance level of <0.01 was used in all analyses. All analyses were carried out using SPSS 20.0.

## Results

### Sample characteristics

A total of 9360 8th and 10th graders (49.2% female) of secondary education were enrolled in in the mandatory sample. In the voluntary sample, a total of 1952 youngsters (55.8% female) participated. All sample characteristics are depicted in Table 1.

**Table 1:** Descriptive statistics per sample

	*n*	Mean (SD)	% of sample		*n*	Mean (SD)	% of sample
Mandatory sample (Twente)				Voluntary sample (IJsselland)			
Age	8761	14.23 (1.14)	-	Age	1571	14.29 (1.07)	-
Education^a^	9295	-	51.5	Education^a^	1723	-	61.3
Gender^b^	9359	-	49.2	Gender^b^	1952	-	55.8
School experiences^c^	9354	2.52 (.85)	-	School experiences^c^	1913	2.13 (.73)	-
Subjective health^d^	9356	1.85 (.70)	-	Subjective health^d^	1892	1.75 (.69)	-
SDQ	9349	-	-	SDQ	1952	-	-
Unlikely	8275	-	88.5	Unlikely	1712	-	91.3
Possible	753	-	8.1	Possible	104	-	5.5
Probable	321	-	3.4	Probable	60	-	3.2
Tobacco consumption^e^	9291	-	9.13	Tobacco consumption^e^	1951	-	3.5
Alcohol consumption^f^	9015	-	51.8	Alcohol consumption^f^	1944	-	26.2
Lifetime alcohol consumption^g^	9280	5.67 (5.26)	-	Lifetime alcohol consumption^g^	1684	2.31 (3.48)	-
Alcohol in past four weeks^h^	9272	-	41.5	Alcohol in past four weeks^h^	1678	-	11.1
Sexual intercourse^i^	9247	1.28 (.79)	-	Sexual intercourse^i^	1649	1.13 (.55)	-

^a^Percentage students HAVO/VWO

^b^Percentage females

^c^(1) great fun, (2) fun, (3) neutral, (4) not fun, (5) dreadful

^d^(1) very good, (2) good, (3) neutral, (4) not good, (5) poor

^e^Percentage daily smoker

^f^Percentage respondents who had ever consumed alcohol

^g^0, 1, 2, 3, 4, 5, 6, 7, 8, 9, 10, 11–19, 20 > times

^h^Percentage respondents who drank alcohol in the past four weeks

^i^(1) never, (2) once, (3) couple of times, (4) regularly

### Comparing demographic characteristics of both samples with population estimates

Findings supported the assumption that voluntary recruiting leads to more selective non-response than mandatory recruiting. A one sample t-test showed that the distribution of gender in the voluntary sample (55.8% female) deviated considerably from available population estimates (48.5% female), received from the CHS of IJsselland, *t*(1951) = 6.038, (*p* < 0.01). Using the population estimates from the CHS of Twente, no significant deviation was found in the mandatory sample regarding gender.

In addition, the estimated mean age for the population of interest in IJsselland [60] was slightly higher (14.5 y) than the age observed in the voluntary sample (14.3 y). Almost no difference was observed in Twente, with an average age of 14.2 years in the estimated population and 14.1 years in the mandatory sample. For education, the discrepancy between the expected proportion of highly educated (HAVO/VWO) students (50.0%) [49] and the observed rates was substantially higher in IJsselland (61.3%) than in Twente (51.5%). Overall, compared to the voluntary sample, the mandatory sample appeared less affected by non-response bias with respect to demographics.

### Effects of sampling bias on demographic characteristics

The average age of participants in the voluntary sample (*M* = 14.29, *SD* = 1.07, *N* = 1571) was not significantly different at the predefined 0.01 level from participants in the mandatory sample (*M* = 14.23, *SD* = 1.14, *N* = 8761; *t*(10,330) = 2.03, *p* = 0.04, two-tailed). The percentage of females in the voluntary sample’s (55.8%) was higher compared to the mandatory sample’s (49.2%; χ^2^(1) = 28.380, *p* < 0.01). For education, the percentage of high education students in the voluntary sample (61.3%) was higher than in the mandatory sample (51.5%; χ^2^(1) = 55.91, *p* < 0.01).

### Effects of sampling bias on health related variables

Bivariate analyses of health related measures revealed several differences between the mandatory sample and the voluntary sample (Table 2). Individuals in the mandatory sample reported worse school experiences (OR = 0.54; 95% CI = 0.50–0.58) and subjective health (OR = 0.80; 95% CI = 0.74–0.86) than individuals in the voluntary sample. Based on the SDQ, a higher prevalence of individuals with a ‘possible’ psychiatric disorder was observed in the mandatory sample (OR = 0.67; 95% CI = 0.54–0.83). No difference was found in the prevalence of a ‘probable’ psychiatric disorder. More participants in the mandatory sample than in the voluntary sample reported daily smoking (OR = 0.37; 95% CI = 0.28–0.47) and having sexual intercourse (OR = 0.71; 95% CI = 0.64–0.78). Regarding alcohol consumption, bivariate odds ratios indicated that more individuals in the mandatory sample had ever consumed alcohol (OR = 0.33; 95% CI = 0.30–0.37). Respondents in the mandatory sample also reported more lifetime alcohol consumption (OR = 0.84; 95% CI = 0.83–0.85) and more recent alcohol use (in the past four weeks) (OR = 0.18; 95% CI = 0.15–0.21). When adjusting for gender, education, and age in multivariable regression analyses (see Table 2) similar odds ratios were found on all health related variables, with 95% confidence intervals largely overlapping in all cases. This indicates that despite controlling for demographic differences, lower tobacco consumption, lower alcohol consumption, better mental health, better subjective health status, more positive school experiences, and less sexual behaviour were found in the voluntary sample compared to the mandatory sample.

**Table 2:** Univariate and multivariable logistic regression analyses

		Univariate model		Multivariable model	
	*n*	OR (95% CI)	Wald	OR (95% CI)	Wald
School experiences^a^	11,267	.54 (.50–.58)^*^	324.36	.62 (.57–.67)^*^	151.51
Subjective health^b^	11,248	.80 (.74–.86)^*^	37.10	.84 (.77–.91)^*^	16.75
SDQ	11,225				
Unlikely ^(ref)^	9987	1.00	14.31	1.00	9.78
Possible	857	.67 (.54–.83)^*^	14.02	.68 (.53–.86)^*^	9.77
Probable	381	.90 (.68–1.20)	.50	1.00 (.72–1.38)	.00
Tobacco consumption^c^	11,242	.37 (.28–.47)^*^	62.22	.45 (.33–.60)^*^	30.08
Alcohol consumption^d^	10,959	.33 (.30–.37)^*^	392.98	.32 (.27–.36)^*^	248.80
Lifetime alcohol consumption^e^	10,964	.84 (.83–.85)^*^	488.22	.81 (.80–83)^*^	388.29
Alcohol in past four weeks^f^	10,950	.18 (.15–.21)^*^	466.79	.13 (.11–.16)^*^	365.73
Sexual intercourse^g^	10,896	.71 (.64–.78)^*^	52.12	.76 (.68–.85)^*^	24.29

Univariate and multivariable logistic regression analyses, separately conducted for each health-related variable versus sampling method (mandatory sample (Twente) = 0; voluntary sample (IJsselland) = 1). All analyses were first conducted without correction for demographic differences between both samples), and then repeated with gender, age, and education level added to the models as covariates

^a^(1) great fun, (2) fun, (3) neutral, (4) not fun, (5) dreadful

^b^(1) very good, (2) good, (3) neutral, (4) not good, (5) poor

^c^Daily smoker: (0) no, (1) yes

^d^Had ever consumed alcohol: (0) no, (1) yes

^e^Lifetime alcohol consumption: 0, 1, 2, 3, 4, 5, 6, 7, 8, 9, 10, 11–19, 20 > times

^f^Had alcohol in the past four weeks: (0) no, (1) yes

^g^(1) never, (2) once, (3) couple of times, (4) regularly

^*^= *p* < .01 (two-tailed)

### Effects of sampling bias on within-subject analyses

Remarkably, no support was found for a moderating role of sampling method on any of the associations between tobacco consumption and one of the following: alcohol consumption, school experiences, mental health, and subjective health status. Similarly, no moderation effects of sampling were found on associations between alcohol consumption and any other health related variables.

## Discussion

The primary aim of this study was to investigate potential effects of non-response bias on prevalence estimates of self-reported health behaviours and well-being, comparing samples obtained from a similar population but with different recruitment strategies and with different non-response ratios. Results showed strong and consistent effects of non-response on all health estimates, as well as considerable effects on the distribution of demographic characteristics. As expected, non-response unambiguously contributed to underestimated health risks.

Expectations derived from literature [6, 15] concerning demographic differences between non-respondents and respondents were confirmed in this study. We found that female, older individuals, and persons with higher education were over-represented in the voluntary sample, while the mandatory sample approached the norm population on these variables. Thus, different sampling methods may recruit different participants, and these demographic differences may be fairly substantial.

Bias due to selective non-response also occurred in health related variables. In line with previous studies [7, 11–27], we found that voluntary respondents report more favourable health indicators, e.g. less smoking, less alcohol consumption, better mental health, better subjective health, more positive school experiences, and less risky sexual behaviour than mandatory respondents. Overall, observed differences between the two samples appeared large to very large, in particular concerning school experiences and alcohol consumption in the past four weeks. For instance, the proportion of respondents who reported alcohol use in the past four weeks was four times higher in the mandatory sample than in the voluntary sample. This is even higher than reported in previous studies regarding alcohol consumption of adults (in which non-response bias was assessed by comparing early and late responders as proxies [61, 62]. Thus, this study indicates that voluntary recruitment may lead to severe underestimation of health-related risk behaviour and mental health problems, compared to mandatory recruitment. Interestingly, this underestimation effect remained highly significant after controlling for the demographic variables. Perhaps being confronted with one’s harmful (smoking), illicit (underage alcohol use), or intimate (sexual behaviour) practices by filling in a survey is perceived as unpleasant (or too private) and motivates these individuals to withdraw from partaking in the survey [8]. These results corroborate recent literature indicating that surveys underestimate risk behaviour due to selective non-response and that this bias increases as response rates fall [28]. Moreover, this study adds that when a controversial topic is involved, motives for not participating are predominantly related to the topic itself, rather than to more generic characteristics [5]. This also implies that in such cases calculating weighted estimates of health related risks to correct for underrepresentation of demographic characteristics would not be sufficient.

Finally, no differences were found between the samples in the strength of the associations between tobacco consumption and alcohol consumption on the one hand and other risk factors on the other. This indicates that non-response did not confound any of these examined associations. Apparently, non-response bias does affect prevalence estimates but within-subject analyses are rather insensitive to such a bias. This may imply that the non-responding boys (or smokers, drinkers, etc.) do not deviate from their responding peers with respect to mechanisms underlying these health-related behaviours in a systematic way. These groups are primarily underrepresented in numbers due to a reluctance to reveal socially undesirable habits. This may have important implications in particular for research on causal mechanisms underlying harmful behaviour and decreased mental health, as a high non-response rate not necessarily poses a threat to the validity of such studies [8].

Clearly, the interpretation of the effect sizes found in this study should be taken with caution as these may depend on the specific characteristics of the samples included in this particular study. Regardless whether these represent small or large effects, however, even small effects may have large impact in public health research. It is argued that the translation of effect size estimates to the assessment of practical importance is not straightforward. Many considerations of the context (e.g. measurement, methodology, and empirical evidence) should be factored into assessments of practical importance [63]. Numerous studies in psychology address important psychological variables or processes, despite the fact that many of them have yielded small effects [64]. Within the context of public health, even small effects in estimates due to non-response bias are relevant.

### Limitations

This study is not without limitations. Two of our central assumptions may not hold, i.e. that (1) there is minimal non-response bias in the mandatory sample and (2) that the true populations in Twente and IJsselland are not intrinsically different. With regard to the first assumption, there is a possibility that youngsters from participating schools in the mandatory sample may not be generalizable to the population of Twente, in spite of the negligible deviations found on demographic characteristics in comparison with population estimates from Twente. The population estimates available may have been insufficient to rigorously test this assumption, as these data have not been published under peer review. The same holds for the IJsselland region. Moreover, in the mandatory sample non-participating schools may differ from participating schools in characteristics relevant to the topic of this study. For instance, non-participating schools may be more likely to be located in deprived neighbourhoods. However, such a bias in the mandatory sample would be likely to contribute to an underestimation of the prevalence of most risk factors within the mandatory sample. This would imply that the true contrast in prevalence estimates between the mandatory and voluntary sample would be even more pronounced than within our current data.

The second assumption, that the adolescent populations in Twente and IJsselland are comparable (as the regions are adjacent, part of the same province, and share similar cultural and topographic characteristics), was partially verified. Two national data sets show a slightly higher smoking prevalence in Twente, and a negligible difference in alcohol consumption among the total population [44–46]. Some caution is needed, as we extrapolated the regional comparisons among the adult population to adolescents. Yet, even when taking this into account, the observed differences in alcohol use and smoking prevalence by far exceed any differences found in both national data sets. For example, the difference in smoking prevalence from the 2012 Health Monitor (23.9% vs. 22.0%) amounts to a relative risk of 1.08, whereas the difference observed between our adolescent samples (9.1% vs 3.5%) equals a relative risk of 2.60. And in the case of alcohol use this contrast is even more distinct. Moreover, the effect sizes found on health related variables remained mostly unchanged when controlling for demographic differences. Therefore, it seems justified to conclude that the consistent underestimation in risk estimates found in this study resulted primarily from non-response bias, and that confounding by true regional differences can only be very small to almost negligible.

Future research may investigate whether our results are replicable in a more controlled design, comparing mandatory and voluntary sampling from an identical population.

## Conclusions

This study is to our knowledge the first to provide direct evidence that the extent of non-response bias in health studies depends on sampling method. Using an identical online survey, a dataset obtained through a mandatory sampling method (school-based) with minimal non-response, was compared to data collected with the more common voluntary sampling method (postal invitation) with presumably a much higher non-response rate. Fortunately, the difference in sampling method did not seem to bias the associations between health-related variables. This suggests that for correlational and longitudinal cohort studies examining within-subject associations between risk factors and health behaviour, non-response bias is not likely to threaten the validity of the results. However, the prevalence of self-reported health variables – tobacco consumption, alcohol consumption, mental health, subjective health status, school experiences, and sexual behaviour - may be substantially underestimated due to selective non-response effects. The large effect sizes we found may have implications for researchers and health policy makers. Researchers should be cautious when recruiting participants for health studies with voluntary recruiting, in particular among adolescents. When the aim is to estimate prevalence or monitor changes over time in prevalence, trends may be missed or mistakenly observed due to non-response bias. And when using voluntary sampling, researchers should employ methods to maximise response rates, and consider data analysis techniques to account for a non-response bias as much as possible. Policy makers should be aware of the likelihood of underestimating adolescent health risks when based on surveys with low response rates.

## Abbreviations

CHS: Community health service
EMOVO: Electronic monitor and health education
OR: Odd ratio
SDQ: Strengths and difficulties questionnaire
VIF: Variance inflation factor
